# Orthopedic Implant-Associated Infection by Multidrug Resistant *Enterobacteriaceae*

**DOI:** 10.3390/jcm8020220

**Published:** 2019-02-08

**Authors:** Bernadette G. Pfang, Joaquín García-Cañete, Julia García-Lasheras, Antonio Blanco, Álvaro Auñón, Raul Parron-Cambero, Alicia Macías-Valcayo, Jaime Esteban

**Affiliations:** 1 Department of Internal Medicine, Hospital Universitario Fundación Jiménez Díaz, IIS-Fundación Jiménez Díaz, UAM, 28040 Madrid, Spain; bernadettepfang@gmail.com (B.G.P.); JCanete@fjd.es (J.G.-C.); Juliagarcialasheras@gmail.com (J.G.-L.); ablancog@fjd.es (A.B.); 2 Department of Orthopaedic Surgery, Hospital Universitario Fundación Jiménez Díaz, IIS-Fundación Jiménez Díaz, UAM, 28040 Madrid, Spain; alvaro.aunon@gmail.com (A.A.); raul.parron@quironsalud.es (R.P.-C.); 3 Department of Clinical Microbiology, Hospital Universitario Fundación Jiménez Díaz, IIS-Fundación Jiménez Díaz, UAM, 28040 Madrid, Spain; alicia.macias@quironsalud.es

**Keywords:** orthopedic implant-associated infection, bone and joint infection, prosthetic joint infection, multidrug resistant *Enterobacteriaceae*, multidrug resistant Gram-negative bacilli

## Abstract

Introduction: Orthopedic implant-associated infections caused by multidrug-resistant *Enterobacteriaceae* are a growing challenge for healthcare providers due to their increasing incidence and the difficulties of medical and surgical treatment. Material and Methods: A retrospective observational study of all cases of multidrug resistant *Enterobacteriaceae* orthopedic implant-associated infection diagnosed in a tertiary European hospital from December 2011 to November 2017 was carried out. Clinical records were reviewed using a previously designed protocol. Data analysis was performed with IBM® SPSS®, version 22. Results: 25 patients met inclusion criteria. The infected implants included 10 prosthetic joints, seven osteosyntheses, six combinations of prosthetic joint and osteosynthesis material, and two spacers. Of the multidrug resistant *Enterobacteriaceae* obtained on culture, 12 were extended-spectrum beta-lactamase-producing *Escherichia coli*, three OXA-48-producing *Klebsiella pneumoniae*, nine extended-spectrum beta-lactamase-producing *Klebsiella pneumoniae*, and one extended-spectrum beta-lactamase-producing *Proteus mirabilis*. Combination antimicrobial therapy was employed in all cases but two. Overall, 16 (64%) patients underwent implant removal. The rate of infection control in the overall implant removal group was 100% compared to 33% in the implant retention group. A strong relationship between implant removal and infection control was observed (*p* = 0.001). Discussion: Implant removal is strongly associated with infection control. However, in some cases, patient age and comorbidity contraindicate hardware extraction. Potential objectives for future studies should be geared towards targeting the population in which debridement, antibiotic therapy, and implant retention can be used as a first-line therapeutic strategy with a reasonable probability of achieving infection control.

## 1. Introduction

The incidence of infections by multidrug resistant *Enterobacteriaceae* (ex Rahn 1937) (MDREB) has increased over the last decades [1,2]. While the prevalence of community acquired MDREB-infection is on the rise, infections are usually nosocomial. Risk factors for infection by these microorganisms, as shown by other reports, include advanced age, previous use of antibiotic treatment, previous hospital admissions, and residing in a long-term-care facility [3]. The most frequent sites of infection are the urinary tract, respiratory system, and surgical wounds [4]. Within this last category, orthopedic device-related infections by MDREB have been described [5], and indeed seem to be gaining importance with an increasing prevalence, as shown from data gathered in recent studies [6]. 

The treatment of bone and joint implant-associated infections by MDREB is a challenge both for clinicians and microbiologists. The complexity of this type of infection stems, not only from the difficulties of designing an effective antimicrobial treatment, which in cases of hardware retention requires combination therapy [7,8], but also from the presence of the implant itself, which favors the formation of biofilms, conditioning difficulty for bacterial eradication [9]. The characteristics of the population at the highest risk for infection by these microorganisms also make them susceptible to adverse effects of long-term antibiotic treatment, which is required in most cases of implant-associated infection. Recently, new drugs have been included in the therapeutic arsenal for the treatment of MDREB infection [10,11], but as of yet, there are no large randomized clinical trials focused on the performance of these drugs in cases of orthopedic implant-associated infection. 

The prognosis of orthopedic device-related infection by MDREB is uncertain. To date, the literature available is scarce, and the few existing reports are small series (three–eight patients), which generally report success rates of less than 50% despite intensive medical and surgical management [12,13], although one series describes a 100% success rate with hardware removal [14].

Our aim is to describe the prevalence of orthopedic implant-associated infections by MDREB over a six-year period in a European tertiary hospital, and to analyze medical and surgical treatment options and results regarding infection control. Secondary outcomes are infection-related patient mortality and sequalae such as residual pain and loss of joint function. 

## 2. Material and Methods

A retrospective analysis of cases of orthopedic device related infections in the Fundación Jiménez Díaz University Hospital, a 686-bed tertiary hospital in Madrid, Spain, during a six-year period (2011–2017) was performed. Out of 482 infections diagnosed and treated by the Bone and Joint Infection Unit, 31 (6.4%) were caused by MDREB. Six patients were excluded from our study as they failed to meet inclusion criteria (presence of orthopedic hardware at the moment of diagnosis, at least one culture positive for MDREB on tissue or hardware samples, and at least six months of follow up post diagnosis, or until death occurred).

MDREB were defined as any enterobacteria resistant to three or more antimicrobial classes [15]. Cases of prosthetic joint infection were defined using the criteria of the International Consensus Group [16]. Other implant-related infections were defined as the presence of clinical signs of infection such as local signs of inflammation, fever, suppuration, and elevation of serum acute phase reactants, with at least one positive culture from surgically obtained tissue or hardware specimens. 

Clinical records were reviewed following a previously determined protocol. Acute infections were considered as those diagnosed less than three months after hardware implantation. The date of diagnosis was considered as the date of the first surgical intervention in which cultures, positive for MDREB, were obtained. Failure was defined as lack of infection control, including persistent signs of infection (fistulae, elevated acute phase reactants), infection-attributable mortality, or the decision to opt for suppressive antibiotic therapy. Information on the functional status of patients at follow-up was also reviewed. 

Continuous variables were expressed as average, range, and median where appropriate, and categorical variables as absolute value and/or percentages of the total sample for that variable. Fisher’s exact test with a two-sided *p* value of less than 0.05 was considered to indicate statistical significance, and to compare averages, Student’s T with a two-sided *p* value of less than 0.05 was used. Data analysis was performed using IBM® SPSS®, version 22.0. Consent to perform the study was obtained from the Research Ethics Committee of our hospital.

## 3. Results

Twenty-five cases of orthopedic implant-associated infection by MDREB were diagnosed between 2011 and 2017 (Table 1). The average age of patients at diagnosis was 74 years (standard deviation (SD): 19; range: 20–90; median: 82). Twenty patients (80%) were female. Five patients (20%) were free from underlying medical conditions at the time of diagnosis. The most frequent comorbid condition was diabetes mellitus (7, 28%) and dementia (7, 28%), followed by obesity (4, 16%), ischemic cardiac disease (4, 16%) and peripheral vascular disease (3, 12%). Eight patients (32%) resided in a long-term care facility. Twenty-two patients (88%) had received antibiotics during the previous six months (10 in the context of another episode of hardware infection), and 16 (64%) had been admitted to hospital at some time over the last six months before diagnosis. Ten patients (40%) had suffered a previous episode of orthopedic implant-associated infection by another microorganism, and 15 of infected hardware (60%) had been implanted during a revision surgery (10 cases as part of surgical treatment of a previous infection, all caused by other microorganisms save one case of chronic femoral osteosynthesis infection in a Syrian refugee, for whom no previous tissue samples had been gathered for culture and who had undergone empiric antibiotic therapy in Syria. This patient was admitted to our Center for a one-step hardware exchange, with bacteremia by Extended-Spectrum Beta-LactamaseESBL producing *Escherichia coli* (Migula 1895) and infection of the new hardware ensuing.) All cases of previous hardware infection took place in the same joint as that infected by MDREB. 

The infected implants included 10 prosthetic joints, seven osteosyntheses, six combinations of prosthetic joint and osteosynthesis material, and two spacers. Twenty acute infections were diagnosed (median time from implantation to diagnosis: 1.2 months; average time: 4 months; standard deviation (SD): 8.5; range: 0-32 months). Clinical manifestations of infection included pain (60%), exudate (56%), erythema (36%), fistula (24%), fever (20%), luxation (16%), and wound dehiscence (8%). Laboratory results showed elevated serum C reactive protein in all cases (average: 14 mg/dL; SD: 15.12; range: 0.6–59.9 mg/dL; upper limit of reference range: 0.5 mg/dL). Leukocytosis was present in cases (average: 8.314 × 10^9^/L; SD: 3.29; range: 3.17–15 × 10^9^/L; reference range: 3.5–11 × 10^9^/L, and neutrophilia in 12 cases (average: 74%; SD: 11.82; range: 38%–93%; reference range: 40%–75%).

Time between the clinical suspicion of infection and the diagnosis of infection ranged between one day and 47 months (median: 2 days; average: 73 days; SD: 292,59). Of the MDREB obtained on culture, 12 were extended-spectrum beta-lactamase-producing *Escherichia coli* (Migula 1895), three OXA-48-carbapenemase-producing *Klebsiella pneumoniae* (Schroeter 1886), nine extended-spectrum beta-lactamase-producing *Klebsiella pneumoniae* (Schroeter 1886), and one extended-spectrum beta-lactamase-producing *Proteus mirabilis* (Hauser 1885). Ten cases were polymicrobial infections. No significant correlation between specific pathogens and outcomes regarding infection control was observed. 

Effective antimicrobial treatment, chosen according to individual susceptibility testing results, was started on an average of 1.8 days from diagnosis (SD: 5.2 days from diagnosis). Combination therapy was started in all cases but two, one in which the patient underwent implant removal as the initial therapeutic strategy, and one in which the patient was assigned to antibiotic suppression before admission to hospital. A carbapenem was used in 23 cases, an aminoglycoside in 15, Fosfomycin in 11, Cotrimoxazole in 5, a cephalosporin in 2, a combination betalactam-betalactamase inhibitor in 2 (1 Ceftazidime-Avibactam, and 1 Amoxicillin-Clavulanic Acid and Cefditoren, the latter as suppressive therapy), Tigecycline in 2 and Doxycycline in 1. No significant association between use of antimicrobials and outcomes was observed. Antibiotic therapy was continued for an average of 54 days (SD: 34.8 days), until the decision to stop antibiotic treatment or opt for suppressive antibiotic therapy was made, or infection-attributable mortality occurred. 

All patients underwent surgical treatment. The initial surgical treatment was debridement, antibiotic therapy and implant retention in 15 cases (2 chronic infections), of which two patients were cured at follow-up without needing further surgical interventions. 13 patients presented initial failure, of which two died within three months of diagnosis (one infection-related death), five patients were assigned to chronic antibiotic suppression therapy, one patient suffered limb amputation, one patient underwent removal of osteosynthesis material, and four patients had a resection arthroplasty. All patients with implant removal (including amputation and resection arthroplasty) achieved infection control. Of the patients assigned to chronic antibiotic suppressive therapy, one of them was admitted to hospital after discharge with signs of implant-related sepsis. An emergency resection arthroplasty was carried out but failed, and the patient died in the early postoperative period. Both patients with chronic infection who were treated with debridement failed to achieve infection control, but implant removal was not performed. 

Ten patients were offered implant removal as the surgical option of choice (four chronic infections). One two-step joint exchange, two spacer removals, four resection arthroplasties, and three removals of osteosynthesis material were carried out. Six patients underwent further surgical treatments to achieve infection control (four patients required at least one further surgical debridement with implant retention, and two patients finally required limb amputation), all of which were successful.

Overall, 16 (64%) patients underwent implant removal. The average age of patients in the overall implant removal group was 69 years (SD: 22.92 years), compared to 83 years (SD: 7.32 years) in the overall implant retention group (T = −2.34, *p* = 0.03). No relationship between the total number of surgical interventions and the probability of failure was observed. Fisher’s exact test was calculated to validate the hypothesis that a correlation existed between implant removal and infection control (*p* = 0.001).

Follow-up was performed for at least 10 months (mean: 32.38 months, SD: 18.19 months, median: 25, 85 months), or until death occurred. Four patients died in the year following diagnosis, two as a direct consequence of infection (1 patient from the debridement and implant retention group, and one in the chronic antibiotic suppression group). At the last visit to the outpatient Orthopedic Surgery clinic, 12 patients were able to walk, of whom 9 had undergone implant removal (five spacer or osteosynthesis removal, three amputation, and one resection arthroplasty). 

## 4. Discussion

MDREBs are a problem of increasing relevance in the field of orthopedic implant-related infections. In line with recent studies on the epidemiology of prosthetic joint infection, which describe an incidence of around 8% for multidrug resistant Gram-negative microorganisms [6], the incidence of MDREB infections in our hospital for the last six years was 6.4%. 

Predisposing conditions were similar to those described in other types of infections by MDREB, principally prior antibiotic treatment, recent hospitalization and residence in a long-term care facility. Seventy-five-percent of cases were acute infections, and more than half of infected hardware had been implanted during revision surgery. These findings underline the predominantly healthcare-associated nature of MDREB infection. 

Combination antibiotic therapy was used in all cases, and no significant difference with regards to outcomes was observed between different groups of antimicrobials. Susceptibility to fluoroquinolones, associated with a higher success rate in cases of implant retention [17], was not shown to improve outcomes in our series. However, the small number of cases is an important limitation.

Regarding surgical treatment, we found a significantly higher rate of infection control in the overall implant removal group compared to the overall implant retention group. The success rate for infection control in the retention group was notably lower than that reported for infections by other microorganisms (33% vs. 47%–68% [17,18,19,20,21]). While two of the failures in this group were chronic infections, and as such, technically not candidates for debridement, antibiotic therapy and implant retention [7], the characteristics of the patients in question (both elderly with important comorbidity) contraindicated more aggressive surgical management. It is of note that, although not statistically significant, the average age of patients in the removal group was lower than that of its counterpart. This may be due to the fact that, in the cases of many elderly patients, treating surgeons opted for conservative management, considering that the risks of surgery outweighed the possible benefits for the patient.

With regards to secondary outcomes such as functional status (measured in ability to walk), the rate of success was higher in the implant removal group. 

While our study is one of the most important series of patients with these infections, the relatively small number of cases, the heterogeneity of the sample with regards to implant, and the observational retrospective nature of the study are important limitations. Potential objectives for future studies should be geared towards targeting the population in which debridement, antibiotic therapy, and implant retention can be used as a first-line therapeutic strategy with a reasonable probability of achieving infection control.

## Figures and Tables

**Table 1:** Case summary.

N	Sex	Age (years)	Implant	Localization	Comorbidity	Long Term Care Facility	Antibiotic Use in Previous 6 Months	Hospitalization in Previous 6 Months	Acute/Chronic	Micro-organism	Polymi-crobial	Initial Surgical Strategy	Final Surgical Treatment	Antibiotic Therapy	Infection Control
1	M	82	PJ	Knee	Chronic coronary disease; Chronic renal disease; COPD	No	Yes	Yes	A	OXA-48 *Klebsiella pneumoniae*	No	DAIR	AST	TGC, SXT and FOX (34 days)	No
2	F	77	PJ	Hip	DM2; Obesity; AF; Dementia	Yes	Yes	No	A	ESBL *Escherichia coli*	No	DAIR	Resection arthroplasty	IPM and FOF (37 days)	Yes
3	F	87	PJ/ OS	Hip	AF	Yes	Yes	Yes	A	ESBL *Escherichia coli*	No	DAIR	AST	IPM and AMK (35 days), and finally FOF as AST	No
4	F	79	OS	Hip	No	No	No	No	A	ESBL *Escherichia coli*	No	DAIR	AST	IPM and SXT (55 days), and finally AST with SXT	No
5	F	83	PJ	Hip	DM2; AF; Chronic coronary disease; dementia	Yes	Yes	Yes	A	ESBL *Escherichia coli*	No	DAIR	Resection arthroplasty	IMP and SXT, posteriorly descaling to SXT and FOF (76 days)	Yes
6	F	84	PJ	Hip	Dementia	Yes	Yes	Yes	C	*ESBL Proteus mirabilis*	Yes	Implant removal	Resection arthroplasty	MEM and AMK (37 days)	Yes
7	F	88	PJ/ OS	Hip	DM2	No	Yes	Yes	A	ESBL *Klebsiella pneumoniae*	No	DAIR	DAIR	MEM and AMK (32 days)	Yes
8	F	78	PJ	Hip	Smoking	Yes	Yes	Yes	A	ESBL *Klebsiella pneumoniae*	No	Implant removal	Two step exchange	MEM and SXT (41 days)	Yes
9	F	66	PJ/OS	Hip	Reumatoid arthritis (under treatment with methotrexate)	No	Yes	Yes	A	ESBL *Klebsiella pneumoniae*	No	DAIR	DAIR	MEM and AMK (48 days)	Yes
10	F	79	PJ	Hip	No	No	Yes	Yes	A	ESBL *Klebsiella pneumoniae*	Yes	DAIR	Resection arthroplasty	IPM and AMK (41 days)	Yes
11	F	86	Sp	Knee	Obesity; Chronic venous insufficiency	No	Yes	Yes	A	ESBL *Klebsiella pneumoniae*	No	Implant removal	Spacer removal and DAIR	MEM, TGC and AMK (44 days)	Yes
12	F	85	PJ/OS	Hip	Dementia	No	Yes	Yes	A	ESBL *Klebsiella pneumoniae*	Yes	DAIR	AST	MEM, FOF and CIP (45 days), finally AST with CDN and AMC	No
13	M	87	PJ	Hip	DM2; AF; Chronic coronary disease	No	Yes	Yes	A	ESBL *Klebsiella pneumoniae*	Yes	Implant removal	Resection arthroplasty	MEM (22 days)	Yes
14	F	90	PJ	Hip	DM2; dementia	No	Yes	Yes	A	OXA-48 *Klebsiella pneumoniae*	No	Implant removal	Resection arthroplasty	AMK and LVX (64 days)	Yes
15	F	73	PJ	Hip	Venous ulcers (calcyphylaxis)	No	Yes	Yes	A	ESBL *Klebsiella pneumoniae*	Yes	DAIR	Resection arthroplasty	AMK and FOF (65 days)	Yes
16	F	30	PJ	Hip	Peripheral vascular disease	No	No	No	C	ESBL *Escherichia coli*	Yes	Implant removal	Amputation	IPM and AMK (124 days)	Yes
17	F	84	Sp	Knee	Chronic coronary disease	Yes	Yes	Yes	A	ESBL *Escherichia coli*	No	Implant removal	Spacer removal and DAIR	MEM and GEN (13 days)	Yes
18	F	85	PJ/ OS	Hip	No	No	Yes	No	C	ESBL *Escherichia coli*	Yes	DAIR	AST	IPM (17 days), finally AST with FOF	No
19	M	86	OS	Femur	DM2; dementia	Yes	Yes	No	A	ESBL *Escherichia coli*	No	DAIR	DAIR	IPM and FOF (29 days)	No
20	F	90	OS	Femur	Dementia	Yes	No	No	A	ESBL *Escherichia coli*	No	DAIR	DAIR	IPM and GEN (44 days)	Yes
21	F	26	OS	Hip	Obesity	No	Yes	No	A	ESBL *Escherichia coli*	No	DAIR	Implant removal	MEM and AMK, later ETP and FOF (61 days)	Yes
22	F	77	OS	Knee	Obesity	No	Yes	No	C	ESBL *Escherichia coli*	Yes	Implant removal	Amputation	MEM and SXT (175 days)	Yes
23	M	20	OS	Knee	No	No	Yes	No	A	ESBL *Escherichia coli*	Yes	DAIR	Amputation	MEM and AMK, later CZA and FOF (76 days)	Yes
24	F	64	OS	Knee	DM2; alcohol abuse	No	Yes	Yes	A	OXA-48 *Klebsiella pneumoniae*	No	Implant removal	Patellectomy	MEM and AMK (74 days)	Yes
25	M	60	OS	Hip	No	No	Yes	Yes	C	ESBL *Klebsiella pneumoniae*	Yes	Implant removal	Implant removal	MEM and AMK (81 days)	Yes

Abbreviations: N: Patient number; M, Male; F, Female; PJ, Prosthetic joint; OS, Osteosynthesis; Sp, Spacer; AF: Atrial fibrillation; DM2, Diabetes Mellitus type 2; A, Acute; C, Chronic; ESBL: Extended-Spectrum Beta-Lactamase; DAIR, Debridement, Antibiotics and Implant Retention; AST, Antibiotic Suppresion Therapy; AMK, amikacin; AMC, amoxicillin-clavulanic acid; CDN, cefditoren; FOX, cefoxitin; CZA, ceftazidime-avibactam; CIP, ciprofloxacin; ETP, ertapenem; FOF, fosfomycin; GEN, gentamicin; IPM, imipenem; LVX, levofloxacin; MEM, meropenem; SXT, trimethoprim-sulfamethoxazole; and TGC, tigecycline.
